# Effects of Combined Inorganic Nitrate and Nitrite Supplementation on Cardiorespiratory Fitness and Skeletal Muscle Oxidative Capacity in Type 2 Diabetes: A Pilot Randomized Controlled Trial

**DOI:** 10.3390/nu14214479

**Published:** 2022-10-25

**Authors:** Kristen D. Turner, Ana Kronemberger, Dam Bae, Joshua M. Bock, William E. Hughes, Kenichi Ueda, Andrew J. Feider, Satoshi Hanada, Luis G. O. de Sousa, Matthew P. Harris, Ethan J. Anderson, Sue C. Bodine, M. Bridget Zimmerman, Darren P. Casey, Vitor A. Lira

**Affiliations:** 1 Department of Health and Human Physiology, College of Liberal Arts and Sciences, University of Iowa, Iowa City, IA 52242, USA; 2 Department of Physical Therapy and Rehabilitation Science, Carver College of Medicine, University of Iowa, Iowa City, IA 52242, USA; 3 Department of Anesthesia, Carver College of Medicine, University of Iowa, Iowa City, IA 52242, USA; 4 Department of Internal Medicine, Carver College of Medicine, University of Iowa, Iowa City, IA 52242, USA; 5 Department of Pharmaceutical Sciences and Experimental Therapeutics, College of Pharmacy, University of Iowa, Iowa City, IA 52242, USA; 6 Fraternal Order of Eagles Diabetes Research Center, University of Iowa, Iowa City, IA 52242, USA; 7 François M. Abboud Cardiovascular Research Center, University of Iowa, Iowa City, IA 52242, USA; 8 Department of Biostatistics, College of Public Health, University of Iowa, Iowa City, IA 52242, USA; 9 Obesity Research and Education Initiative, University of Iowa, Iowa City, IA 52242, USA

**Keywords:** mitochondria, oxygen consumption, nutraceutical

## Abstract

Nitric oxide (NO) stimulates mitochondrial biogenesis in skeletal muscle. However, NO metabolism is disrupted in individuals with type 2 diabetes mellitus (T2DM) potentially contributing to their decreased cardiorespiratory fitness (i.e., VO_2_max) and skeletal muscle oxidative capacity. We used a randomized, double-blind, placebo-controlled, 8-week trial with beetroot juice containing nitrate (NO_3_^−^) and nitrite (NO_2_^−^) (250 mg and 20 mg/day) to test potential benefits on VO_2_max and skeletal muscle oxidative capacity in T2DM. T2DM (N = 36, Age = 59 ± 9 years; BMI = 31.9 ± 5.0 kg/m^2^) and age- and BMI-matched non-diabetic controls (N = 15, Age = 60 ± 9 years; BMI = 29.5 ± 4.6 kg/m^2^) were studied. Mitochondrial respiratory capacity was assessed in muscle biopsies from a subgroup of T2DM and controls (N = 19 and N = 10, respectively). At baseline, T2DM had higher plasma NO_3_^−^ (100%; *p* < 0.001) and lower plasma NO_2_^−^ levels (−46.8%; *p* < 0.0001) than controls. VO_2_max was lower in T2DM (−26.4%; *p* < 0.001), as was maximal carbohydrate- and fatty acid-supported oxygen consumption in permeabilized muscle fibers (−26.1% and −25.5%, respectively; *p* < 0.05). NO_3_^−^/NO_2_^−^ supplementation increased VO_2_max (5.3%; *p* < 0.01). Further, circulating NO_2_^−^, but not NO_3_^−^, positively correlated with VO_2_max after supplementation (R^2^= 0.40; *p* < 0.05). Within the NO_3_^−^/NO_2_^−^ group, 42% of subjects presented improvements in both carbohydrate- and fatty acid-supported oxygen consumption in skeletal muscle (vs. 0% in placebo; *p* < 0.05). VO_2_max improvements in these individuals tended to be larger than in the rest of the NO_3_^−^/NO_2_^−^ group (1.21 ± 0.51 mL/(kg*min) vs. 0.31 ± 0.10 mL/(kg*min); *p* = 0.09). NO_3_^−^/NO_2_^−^ supplementation increases VO_2_max in T2DM individuals and improvements in skeletal muscle oxidative capacity appear to occur in those with more pronounced increases in VO_2_max.

## 1. Introduction

Type 2 Diabetes Mellitus (T2DM) affects over 460 million people worldwide [1] and is associated with a significant increased risk of cardiovascular disease and all-cause mortality [2,3]. Cardiorespiratory fitness (i.e., VO_2_max), which is inversely associated with cardiovascular disease and all-cause mortality risks [4], is decreased by ≥15% in T2DM patients [5,6]. Therefore, strategies that increase VO_2_max may mitigate morbidity and mortality in T2DM patients. Regular exercise is an effective intervention that can increase VO_2_max in T2DM patients along with improvements in insulin sensitivity and glucose metabolism [7,8,9]. However, substantial reductions in VO_2_max invariably cause simple daily living activities to become more physically demanding, which make it more challenging for individuals to engage in exercise programs. In fact, 39% of adults with diabetes report being physically active compared to 58% of those without diabetes [10]. Collectively, these observations indicate that additional strategies to improve VO_2_max should have significant clinical implications in T2DM.

VO_2_max is dependent on three main factors, namely respiratory mechanics and alveolar gas exchange, delivery of oxygen to the working muscle via blood flow and utilization of oxygen by muscle mitochondria [11]. Among these, limitations in muscle blood flow and oxygen utilization have been extensively documented in T2DM patients [12,13,14,15]. Of note, however, the bioavailability of the signaling molecule nitric oxide (NO) is decreased in animal models of diabetes as well as in diabetic humans [16,17]. This is relevant because NO has been shown not only to regulate blood flow to contracting muscles, but also to stimulate muscle mitochondrial biogenesis [18,19,20,21]. The L-arginine-NO synthase (NOS) pathway is a major contributor to NO formation in mammals. Once synthesized, NO is rapidly oxidized to form nitrite (NO_2_^−^) and nitrate (NO_3_^−^). However, the conversion of NO_3_^−^ and NO_2_^−^ back to NO also occurs in blood and various tissues [22,23,24,25] and NO_3_^−^ reduction (i.e., nitrate-nitrite-NO pathway) represents an alternative pathway for generation of NO [25]. In a recent related study, we demonstrated that the combined supplementation of inorganic NO_3_^−^ and NO_2_^−^ increases NO bioavailability in T2DM [26]. Here, we hypothesized that combined supplementation of inorganic NO_3_^−^ and NO_2_^−^ would improve VO_2_max and skeletal muscle mitochondria respiratory function in T2DM participants. We report findings in T2DM participants and age- and BMI-matched non-diabetic controls and address our central hypothesis using a double-blinded, placebo-controlled trial. We have also explored associations between circulating NO_3_^−^ and/or NO_2_^−^ with VO_2_max in T2DM to determine the extent to which these NO-related metabolites or precursors may predict outcomes of the supplementation.

## 2. Materials and Methods

### 2.1. Ethical Approval

Patients provided written, informed consent prior to participating in experimental protocols approved by the University of Iowa’s Institutional Review Board. Data reported in this manuscript were collected in accordance with the principles of the Declaration of Helsinki during a registered clinical trial (ClinicalTrials.gov ID: NCT02804932) with some related data previously published. Specifically, baseline data from 30/36 patients in the present study were compared to 15 controls without T2DM to identify if peripheral α-mediated vasoconstriction is exaggerated in patients with T2DM [15]. Demographical, along with pre-intervention endothelium-dependent vasodilation data, were published to examine the relationship between microvascular endothelial function and glycemic management [27]. Impact of the intervention on forearm skeletal muscle perfusion to handgrip exercise and blood pressure in T2DM patients was examined elsewhere [26,28].

### 2.2. Study Participants

Age- and BMI-matched T2DM and non-diabetic control participants were enrolled in the study from 2016 to 2019. Exclusion criteria for all subjects included age of ≤40 and ≥77 years, BMI of ≥42 kg/m^2^, cardiovascular events in the prior year (e.g., heart attack, stroke), heart failure, symptomatic coronary artery disease, hypotension (i.e., resting systolic BP < 90 mmHg), renal impairment with creatinine clearance (estimated glomerular filtration rate of <50 mL/min), current tobacco use or recent (<1 yr.) cessation, use of anti-coagulant drugs, use of medication containing nitrates or participation in research studies in which medications or interventions that could alter subject responses in the current study. T2DM patients were included if their diagnosis (along with their medications) was corroborated via review of electronic medical records. Control subjects were examined at baseline and T2DM patients were examined at baseline and after an 8-week, double-blinded, placebo-controlled intervention with beetroot juice. The total number of T2DM patients participating in the intervention was based on a priori sample size calculation related to a different outcome measure of the larger clinical trial (i.e., muscle blood flow) [26].

### 2.3. Maximal Exercise Testing

A 12-lead electrocardiogram (ECG), symptom limited cardiopulmonary exercise testing with gas exchange measurements (Parvo Medics, Sandy, UT, USA) was performed on a cycle ergometer (Lode Corival Bike Ergometer, Groningen, The Netherlands) using a ramp protocol to determine cardiorespiratory fitness (VO_2_max). The rate of perceived exertion (RPE), heart rate and brachial blood pressure were measured throughout the test and all tests were monitored by a physician. The exercise protocol consisted of a two-minute warmup with no resistance. The first minute was performed at 20 W and then increased by 10 W every minute thereafter until volitional fatigue. Maximal effort was defined as achieving 90% age-predicted maximal heart rate (i.e., 220-age), a respiratory exchange ratio (RER) ≥ 1.1 and a RPE ≥ 17 with VO_2_max defined as the average of the two or three highest measurements (each reflecting 15-sec average data points) during the final minute of exercise. Maximal work rate (WRmax) was defined as the highest work rate (in Watts) achieved during exercise test.

### 2.4. Nitrate (NO_3_^−^)/Nitrite (NO_2_^−^) Supplementation

As described previously [26], patients were randomly assigned in a double-blinded parallel fashion to consume either beetroot juice containing 250 mg NO_3_^−^ (4.0 mmol) and 20 mg NO_2_^−^ (0.3 mmol), which is comparable to 100 g of red beetroot or fresh spinach [29], or ~20 mg NO_3_^−^ (~0.08 mmol) without any NO_2_^−^ (placebo) daily for eight weeks. Beetroot beverages consisted of beetroot powder (Superbeets, HumanN, Inc., Austin, TX, USA) dissolved in 4–6 ounces of water. The NO_3_^−^ and NO_2_^−^ content of both supplements was verified via high-performance liquid chromatography prior to the start of data collection. Participants were instructed to consume those once per day, before the first meal in the morning. Two subjects reported gastrointestinal discomfort with the beetroot juice consumption and were asked to consume the beverage two hours after their first meal. All subjects were instructed to adhere to a low-nitrate diet (e.g., no leafy vegetables or processed meats) 48 h prior to study visits. Given the anti-bacterial properties of mouthwash [30], subjects were also instructed not to use these products before beetroot juice consumption and for two hours afterwards. Post study visits were conducted 18–24 h following consumption of the supplements.

### 2.5. Blood Sampling and Muscle Biopsies

The blood collection and muscle biopsies were performed 48–72 h apart from maximal exercise testing. Blood was collected in tubes containing EDTA. Samples were centrifuged, aliquoted and frozen at −80 °C until further analysis, as described [26]. Plasma NO_3_^−^ and NO_2_^−^ were determined by chemiluminescence (NOA 280i, Sievers Instruments, Boulder, CO, USA). In addition, quantification of fasting blood glucose, insulin and HbA1c were performed at the University of Iowa Hospitals and Clinics using standard laboratory protocols [26].

Vastus lateralis muscle biopsies (~100–150 mg) were obtained with a percutaneous needle using the Bergstrom technique under local anesthesia. All study participants who received a muscle biopsy and/or had blood drawn did so after an overnight (minimum of eight hours) fasting period, refraining from caffeine and alcohol. While physical activity was not monitored throughout the study, all subjects were instructed to refrain from exercise for at least 24 h prior to the study visit. In addition, all subjects withheld their medication(s) the morning of the biopsy. The final number of muscle biopsies provided and analysis performed for each sample, was determined by: (a) number of subjects that consented to the biopsy; and (b) amount of fat contamination from intramuscular stores which at times limited the amount of muscle obtained and the variables that could be reliably assessed from those.

### 2.6. Histology and Fluorescence Microscopy

Approximately 20 mg of tissue obtained from biopsies were immediately embedded in tissue-freezing medium for subsequent immunofluorescence analysis as described [31]. Serial sections of muscle samples (10 μm) were obtained at a temperature of −24–25 °C. For fiber type distribution, sections were stained with specific antibodies for Laminin (Sigma L9393, 1:500), myosin heavy chain (MyHC) type 1 (MYH7 (type 1)—BA-F8), type 2A (MYH2 (Type 2A)—SC-71) and type 2X (MYH1 (type 2X)—6H1). These were from Developmental Studies Hybridoma Bank and were used at 1:250 dilution, as previously described [32]. Samples were mounted using appropriate medium (Prolonged Diamond or Glass; Invitrogen, # P36970 or P36980) and images were obtained via confocal microscopy (Zeiss LSM710; Jena, Germany). An average of 384 fibers was analyzed per section and all histological analyses described were completed in a single-blinded fashion using ImageJ (NIH).

### 2.7. Skeletal Muscle Oxidative Capacity

Skeletal muscle oxidative capacity, also referred to as mitochondrial respiratory capacity, was assessed as previously described [33,34,35,36]. Approximately 50 mg of skeletal muscle tissue obtained from the biopsy sample was immediately placed in ice cold preservation buffer (7.23 mM K_2_EGTA, 2.77 mM CaK_2_EGTA, 20 mM imidazole, 0.5 mM DTT, 20 mM taurine, 5.7 mM ATP, 14.3 mM phosphocreatine, 6.56 mM MgCl_2_·6H_2_O and 50 mM MES; (pH 7.1, 295 mosmol/kgH_2_O)). Muscle fibers were gently separated under a light microscope and fiber bundles were then permeabilized in preservation buffer with 50 μg/mL of saponin for 30 min. Permeabilized fiber bundles were transferred to ice cold respiration buffer (105 mM K-MES, 30 mM KCl, 1 mM EGTA, 10 mM K_2_HPO4, 5 mM MgCl_2_·6H_2_O, 20 µm blebbistatin and 2.5 mg/mL bovine serum albumin (BSA, pH 7.4, 290 mosmol/kgH_2_O)) and remained on ice until high-resolution respirometry (OROBOROS Instruments, Innsbruck, Austria) was performed. For carbohydrate (CHO)-supported respiration, fiber bundles (3–4 mg) were used to assess respiration supported with pyruvate (5 mM) and malate (2 mM) with subsequent addition of ADP (5 mM) and glutamate (5 mM). Maximal CHO-supported respiratory capacity was determined in the presence of these substrates with subsequent addition of succinate (5 mM). Subsequently, respiration was assessed under Complex I and Complex V (i.e., ATP synthase) inhibition with the use of Rotenone (0.01 mM) and Oligomycin (10 μg/mL), respectively. Fatty acid (FA)-supported respiration was assessed in separate fiber bundles (also 3–4 mg) exposed to palmitoyl-carnitine (0.075 mM) and malate (1 mM). Maximal FA-supported respiratory capacity was determined in the presence of these substrates with subsequent addition of ADP (5 mM). Assessments of mitochondrial respiratory capacity were performed in a blinded fashion throughout the NO_3_^−^/NO_2_^−^ intervention.

### 2.8. Statistical Analyses

Data is reported as means ± standard deviation for the primary group (i.e., all individuals participating in the study) and sub-group (i.e., individuals from whom muscle biopsies were obtained). Two-sample *t*-tests were used for continuous variable comparisons, whereas Chi-square tests were used for categorical variable comparisons between non-diabetic controls and T2DM participants before the intervention. For examination of potential benefits of combined NO_3_^−^/NO_2_^−^ supplementation, individual delta changes for each dependent variable were calculated for each group (i.e., placebo and NO_3_^−^/NO_2_^−^ supplementation). Then, an Analysis of Covariance (ANCOVA) was used to test differences for each dependent variable with pre-intervention values for that same variable as a covariate. This approach allowed the comparison of the main effects of the intervention while controlling for potential baseline differences between placebo and NO_3_^−^/NO_2_^−^ supplemented subjects that might have originated from random group assignments. Due to the lower number of subjects providing muscle biopsies, ANCOVA was generally underpowered in this subgroup and additional analyses (e.g., within group paired t test) were conducted to identify potential trends originating from the intervention. Chi-square tests were also used to compare the proportion of participants improving mitochondrial respiration or not under CHO- and FA-supported conditions among those in the NO_3_^−^/NO_2_^−^ supplementation and placebo groups. Pearson’s partial correlation coefficients and related coefficients of determination (R^2^) were established between plasma NO_3_^−^ or NO_2_^−^ levels and VO_2_max after supplementation. Grubb’s test identified one subject of the placebo sub-group as an outlier due to atypical changes in mitochondrial respiration during the intervention. Their data was excluded from such analyses. Statistical significance was determined a priori at *p* < 0.05. ANCOVA analyses were performed using SPSS (version 27.0; IBM Corp., Armonk, NY, USA), whereas all other analyses were conducted using Prism (version 9.0.0 (121); GraphPad Software, San Diego, CA, USA).

## 3. Results

### 3.1. Baseline Characteristics

**Figure 1:**
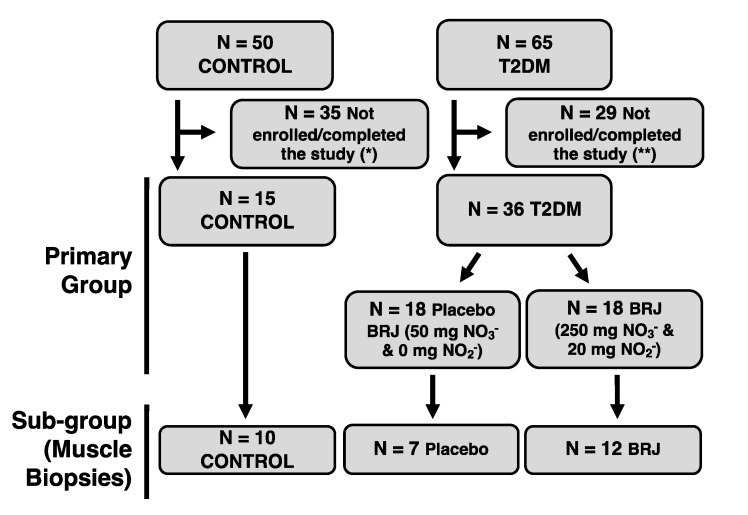
Consort diagram. Diagram illustrating enrollment of patients with type 2 diabetes mellitus (T2DM) and non-diabetic control participants for the entire study (Primary group). The number of participants that provided muscle biopsies (Sub-group) is also shown. (*) Non-diabetic control subjects that did not participate in the study included 11 subjects who did not qualify based on screening questionnaire (either because of too much regular exercise, specific medications, or other studies they were enrolled in), 2 subjects because bifurcation of their brachial artery was identified via Doppler examination and 22 subjects that were either out of contact or did not match to our diabetic population. (**) T2DM patients that did not participate in the study included 5 subjects because of medications used, 1 subject because of chronic kidney disease, 1 subject because of current smoking status, 1 subject misdiagnosed with T2DM, 2 subjects because their body mass index was too high, 14 subjects out of contact or declining to participate prior to consenting, 4 subjects who did not complete the entire study and 1 subject who did not reach VO_2_max in one of the exercise capacity evaluations. The 36 T2DM patients enrolled in the study were assigned via random number generator to receive either placebo or nitrate/nitrite supplementation with a one-to-one allocation. Note: the final number of participants studied with muscle biopsies was influenced by: (i) subjects not consenting to the procedure; or (ii) technical problems occurring in at least one of the testing visits (ex: not enough muscle tissue and/or extensive proportion of intramuscular fat obtained). Note: BRJ = beetroot juice.

T2DM patients (N = 65) and non-diabetic controls (N = 50) were originally screened. Thirty-six (36) patients diagnosed with T2DM (primary group of T2DM patients) and 15 control subjects (primary group of controls) signed consents and completed the study (Figure 1). Muscle biopsies were obtained in 10 of the 15 control subjects (sub-group of controls) and 19 of the 36 T2DM participants (sub-group of T2DM). All clinical baseline characteristics of primary groups and sub-groups are shown in Table 1.

#### 3.1.1. Clinical Characteristics and Medications

As expected, only T2DM subjects were on medications for glycemic control and had elevated blood glucose and HbA1c levels. In addition, a higher proportion of T2DM subjects vs. non-diabetic controls were taking statins and angiotensin receptor blockers (72 vs. 33% and 25 vs. 0%, respectively). Medications used by participants in the placebo and NO_3_^−^/NO_2_^−^ supplementation groups were comparable. Clinical characteristics and medications used by non-diabetic controls and T2DM subjects in the sub-group that provided muscle biopsies were consistent with those observed in the original primary sample, except that the reported duration of T2DM was higher among those in the NO_3_^−^/NO_2_^−^ group vs. placebo.

#### 3.1.2. Plasma NO Metabolites, VO_2_max and Work Rate Capacity in T2DM and Non-Diabetic Controls

As shown in Table 2, T2DM patients had higher plasma NO_3_^−^ both in the primary group and sub-group when compared to non-diabetic controls (100% and 117.5%, respectively; *p* < 0.001). Conversely, plasma NO_2_^−^ levels were lower in the T2DM patients in both the primary group (−46.8%; *p* < 0.0001) and sub-group (−42.0%; *p* < 0.001). VO_2_max was decreased in the primary group of T2DM patients in comparison to non-diabetic controls and this deficit was preserved among participants that provided muscle biopsies (−26.4% and −29.3%, respectively; *p* < 0.01). Similarly, the maximal individual work rates of T2DM patients were lower in the primary group and sub-group in relation to non-diabetic controls (−23.6% and −30.1%, respectively; *p* < 0.01).

#### 3.1.3. Skeletal Muscle Fiber Types and Oxidative Capacity

**Figure 2:**
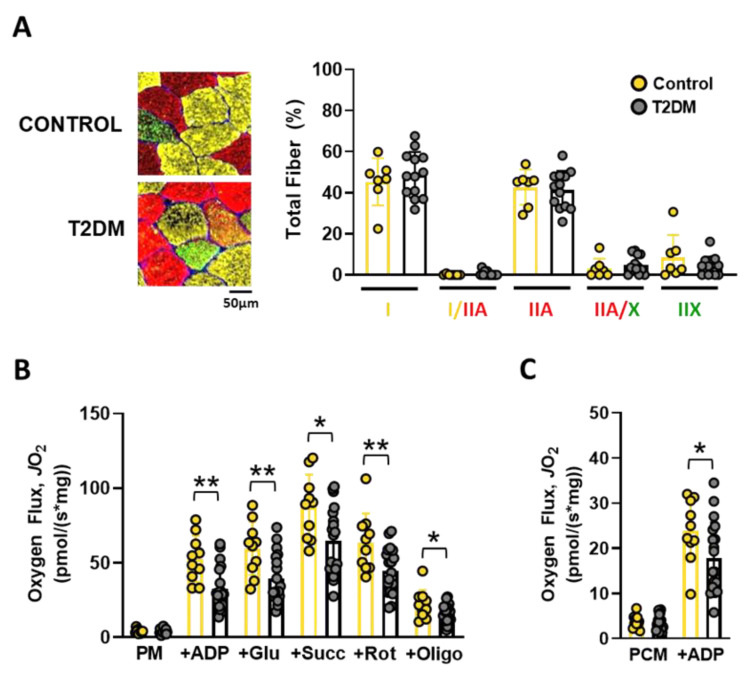
Muscle fiber type distribution and mitochondrial respiratory capacity in skeletal muscle of T2DM and non-diabetic controls. (**A**) (Left) Cross-sections of skeletal muscle biopsies denoting fibers expressing MyHC I (type I fibers, yellow), MyHC IIA (type IIA fibers, red) and MyHC IIX (Type IIX fibers, green). (Right) Quantification of fiber type distribution. (**B**) Quantification of carbohydrate-supported mitochondrial respiration in permeabilized muscle fibers of control (N = 10) and T2DM (N = 19). (**C**) Fatty-acid supported mitochondrial respiration in permeabilized muscle fibers of control (N = 10) and T2DM (N = 19). Data are presented as mean ± SD. * *p* < 0.05; ** *p* < 0.01.

Fiber type distribution was comparable between T2DM participants and non-diabetic controls (Figure 2A). Citrate synthase protein levels, as a surrogate of mitochondrial content, were also similar between T2DM participants and controls (Supplemental Figure S1). CHO-supported basal respiration (pyruvate with malate) was not different between controls and T2DM participants. However, ADP-stimulated respiration was decreased in T2DM by 37.8% (95% CI: −31.76, −8.052; *p* = 0.019). Similar decreases were observed in T2DM after the subsequent additions of the TCA cycle substrates glutamate (−33.9%; 95% CI: −33.66, −6.777; *p* = 0.0046) and succinate (i.e., maximal CHO-supported respiration, −26.1%; 95% CI: −40.63, −5.025; *p* = 0.0139). Interestingly, these decreases persisted after inhibition of the electron transport chain (ETC) complexes I with rotenone (−29.8%; 95% CI: −32.47, −5.358; *p* = 0.008) and V (a.k.a. ATP synthase) with oligomycin (−27.7%; 95% CI: −12.07, −0.1167; *p* = 0.046), indicating a broad impairment in mitochondrial respiratory capacity, rather than deficiencies in specific ETC complexes (Figure 2B). Accordingly, maximal FA-supported respiration (i.e., palmitoyl-carnitine with malate and ADP) was also impaired in T2DM muscles (−25.5%; 95% CI: −12.17, −0.0293; *p* = 0.049) (Figure 2C).

### 3.2. Effects of Combined NO_3_^−^/NO_2_^−^ Supplementation

Combined NO_3_^−^/NO_2_^−^ supplementation did not alter BMI and variables associated to glycemic control in T2DM in the primary group [26] or sub-groups of subjects (Supplemental Table S1). In addition, no changes in medication occurred during the 8-week intervention in either the placebo or NO_3_^−^/NO_2_^−^ groups.

#### 3.2.1. Plasma NO Metabolites

**Figure 3:**
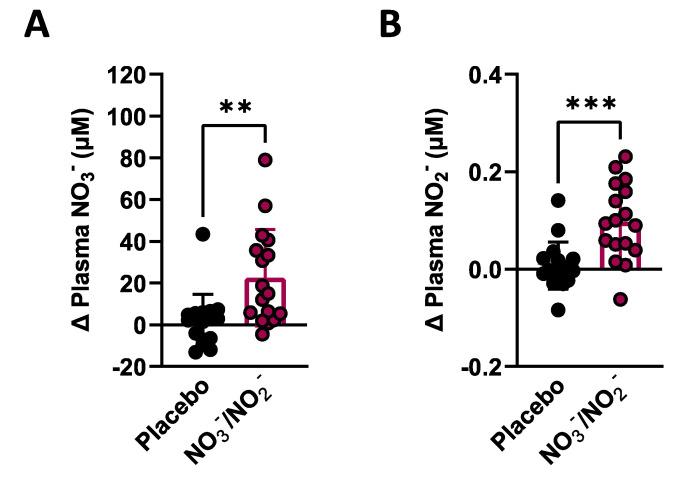
Plasma nitrate (NO_3_^−^) and nitrite (NO_2_^−^) levels after combined NO_3_^−^/NO_2_^−^ supplementation vs. Placebo in T2DM participants. (**A**) ANCOVA results, using pre-supplementation values of NO_3_^−^ as a covariate, showing individual changes (post–pre) for plasma NO_3_^−^ from primary group of T2DM subjects (N = 18/condition). (**B**) ANCOVA results, using pre-supplementation values of NO_2_^−^ as a covariate, showing individual changes (post–pre) for plasma NO_2_^−^ as shown in A. Data are presented as mean ± SD. ** *p* < 0.01; *** *p* < 0.001.

As previously reported [26], significant increases in plasma levels of NO_3_^−^ (mean difference between NO_3_^−^/NO_2_^−^ and placebo groups of 20.6 µmol [95% CI: 7.56, 33.56]; *p* < 0.0029) and NO_2_^−^ (mean difference between NO_3_^−^/NO_2_^−^ and placebo groups of 0.090 µmol [95% CI: 0.045, 0.136]; *p* < 0.0002) were observed with the NO_3_^−^/NO_2_^−^ supplementation over placebo (Figure 3A,B). Equivalent increases were observed in the group of participants that provided muscle biopsies, with both plasma levels of NO_3_^−^ (mean difference between NO_3_^−^/NO_2_^−^ and placebo groups of 26.8 µmol [95% CI: 5.35, 48.31]; *p* < 0.0173) and NO_2_^−^ (mean difference between NO_3_^−^/NO_2_^−^ and placebo groups 0.102 µmol [95% CI: 0.040, 0.164]; *p* < 0.0028) being significantly increased with NO_3_^−^/NO_2_^−^ supplementation over placebo (Supplemental Figure S2A,B).

#### 3.2.2. VO_2_max and WRmax

**Figure 4:**
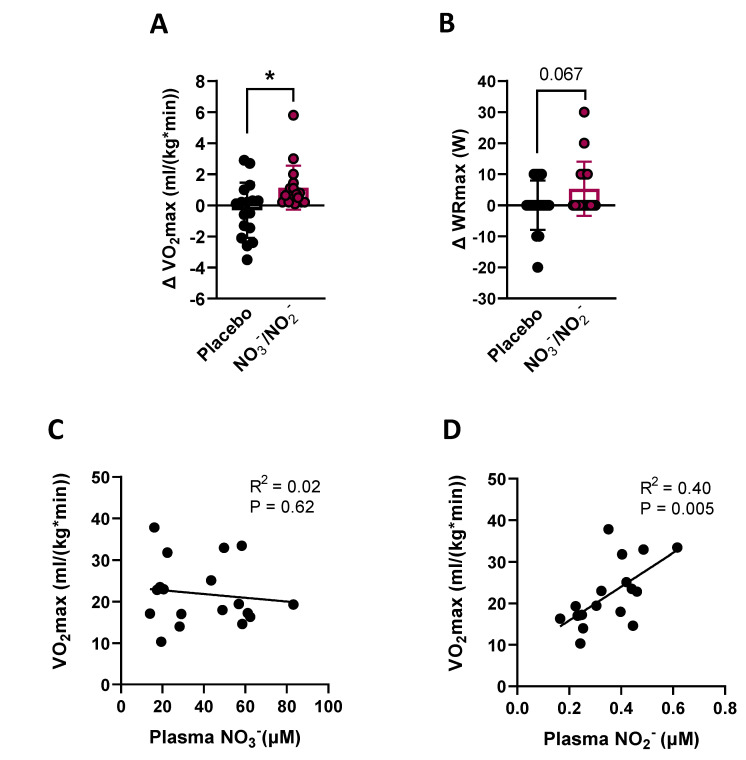
Combined nitrate/nitrite (NO_3_^−^/NO_2_^−^) supplementation increases maximal oxygen uptake capacity (VO_2_max) in T2DM participants. (**A**) ANCOVA results, using pre-supplementation values of VO_2_max as a covariate, showing individual changes (post–pre) for VO_2_max from primary group of T2DM subjects (N = 18/condition). (**B**) ANCOVA results, using pre-supplementation values of maximal work rate (WRmax) as a covariate, showing individual changes (post–pre) for WRmax from primary group of T2DM subjects (N = 18/condition). (**C**) Relationship between plasma NO_3_^−^ and VO_2_max after NO_3_^−^/NO_2_^−^ supplementation (N = 18; r = 0.14; Slope 95% CI: −0.237, 0.146). (**D**) Relationship between NO_2_^−^ and VO_2_max after NO_3_^−^/NO_2_^−^ supplementation (N = 18; r = 0.63; Slope 95% CI: 14.09, 67.34). Data are presented as mean ± SD. * *p* < 0.05.

NO_3_^−^/NO_2_^−^ supplementation significantly increased VO_2_max by approximately 5.3%, with no effect of placebo in the primary group of participants (mean difference between NO_3_^−^/NO_2_^−^ and placebo groups of 1.45 mL/(kg*min) [95% CI: 0.33, 2.56]; *p* < 0.0264) (Figure 4A). WRmax also seemed to improve with NO_3_^−^/NO_2_^−^ supplementation (mean difference between NO_3_^−^/NO_2_^−^ and placebo groups of 5.3 W [95% CI: −0.53, 11.12]; *p* = 0.067) (Figure 4B). ANCOVAs did not detect significant changes in VO_2_max or WRmax in the smaller number of participants that provided muscle biopsies (Supplemental Figure S3A,C). However, additional within group pre- vs. post-supplementation t tests demonstrated clear trends for positive changes in VO_2_max and WRmax with NO_3_^−^/NO_2_^−^ supplementation in this sub-group (Supplemental Figure S3B,D).

Changes in plasma NO_3_^−^ and/or NO_2_^−^ resulting from supplementation were not correlated with changes in VO_2_max. However, plasma NO_2_^−^, but not NO_3_^−^, was significantly correlated with VO_2_max after supplementation in both the primary group (r = 0.63, R^2^ = 0.40; *p* = 0.005) and sub-group of participants (r = 0.64, R^2^ = 0.40; *p* = 0.036) (Figure 4C,D and Supplementary Figure S3D,F).

#### 3.2.3. Skeletal Muscle Oxidative Capacity

**Figure 5:**
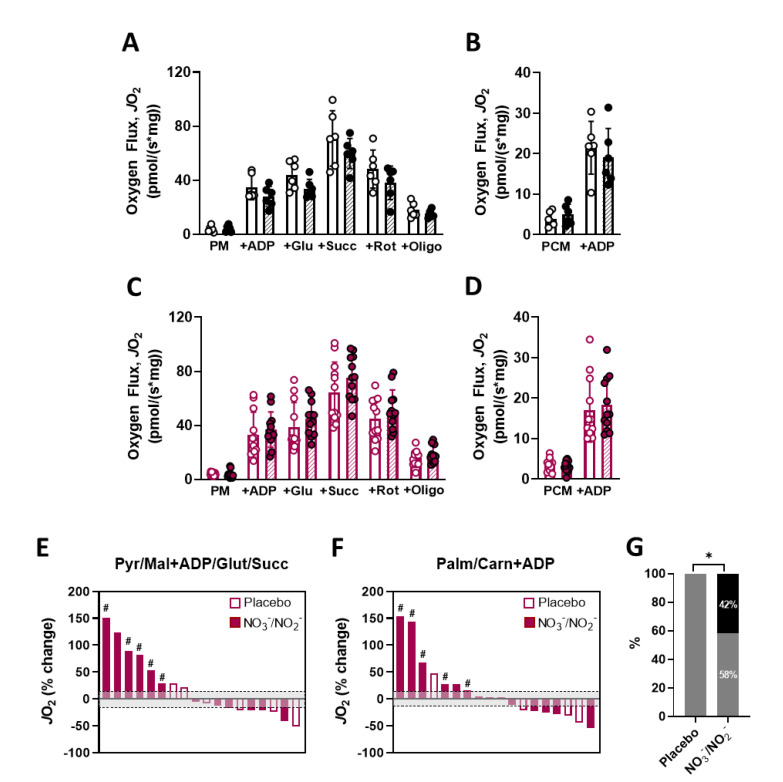
Mitochondrial respiratory capacity in skeletal muscle of T2DM after combined NO_3_^−^/NO_2_^−^ supplementation. (**A**) Quantification of carbohydrate (CHO)-supported mitochondrial respiration in permeabilized muscle fibers of T2DM in the placebo group (N = 6). (**B**) FA-supported mitochondrial respiration in the same conditions as described in A. (**C**) Quantification of CHO-supported mitochondrial respiration in permeabilized muscle fibers of T2DM in the NO_3_^−^/NO_2_^−^ group (N = 12). (**D**) Fatty Acid (FA)-supported mitochondrial respiration in the same conditions as described in C. (**E**) Waterfall plot denoting individual % changes in CHO-supported mitochondrial respiration after intervention in both placebo and NO_3_^−^/NO_2_^−^ groups. Shaded area represents CV of high resolution respirometry in permeabilized human muscle fibers [37]. (**F**) Waterfall plot denoting individual % changes in FA-supported mitochondrial respiration as described in E. (**G**) Percentage of individuals that presented increases in both CHO-supported and FA-supported respiration (black section of columns) vs. others (grey section of columns). Data are presented as mean ± SD for A, B, C and D. Common subjects in Figure 5E,F (i.e., highly responsive to the supplementation in terms of mitochondrial respiration) are denoted by “#”. * *p* < 0.05.

Mean group values of CHO- and FA-supported respiration did not significantly change with NO_3_^−^/NO_2_^−^ supplementation or placebo (Figure 5A–D). We did not also observe significant differences with NO_3_^−^/NO_2_^−^ supplementation in relation to placebo via ANCOVA analyses (*p* = 0.16 and *p* = 0.39, respectively, for maximal CHO- and FA-supported respiration; Supplementary Figure S4A,B). ANCOVA analysis also did not reveal changes in citrate synthase protein levels between NO_3_^−^/NO_2_^−^ supplementation and placebo, but pre- vs. post-supplementation t test analyses showed trends for increases in CS protein with NO_3_^−^/NO_2_^−^ supplementation (Supplementary Figure S4C–E). We then performed additional qualitative analyses comparing the proportion of individuals in placebo and NO_3_^−^/NO_2_^−^ groups that presented increases in mitochondrial respiration above 15.3%, which has been reported as the coefficient of variation for high resolution respirometry of permeabilized human muscle fibers [37]. Interestingly, the proportion of subjects that presented improvements in both CHO- and FA-supported respiration was 42% (5 of 12) after NO_3_^−^/NO_2_^−^ supplementation vs. 0% receiving placebo (*p* < 0.05; Figure 5E–G). We considered these subjects as highly responsive to the intervention for skeletal muscle oxidative capacity. Despite the small N, this highly responsive sub-sample tended to present higher VO_2_max increases than the rest of the NO_3_^−^/NO_2_^−^ group that provided biopsies (*p* = 0.09; Supplementary Table S2).

## 4. Discussion

Here, we extend previous findings [5,6] and demonstrate that T2DM patients had decreased VO_2_max (approx. −26%) in comparison to age- and BMI-matched non-diabetic controls. Contrary to select previous reports [38,39], these differences were not associated with a decreased percentage of slow twitch muscle fibers (MyHC I) (Figure 2A) or mitochondrial content (as indicated by comparable skeletal muscle CS expression) between T2DM and controls (Supplementary Figure S1). Although other mitochondrial proteins could have been decreased in T2DM muscles, collectively our results suggest that most of the deficit in skeletal muscle oxidative capacity observed in T2DM patients was due to dysregulation/dysfunction of mitochondria rather than its decreased content. It is also well established that T2DM patients present decreased NO bioavailability [17]. In fact, to our knowledge this is the first study reporting beneficial effects of combined NO_3_^−^/NO_2_^−^ supplementation on VO_2_max and skeletal muscle oxidative capacity in T2DM patients. The observed 5.3% increase in VO_2_max resulting from this nutraceutical or dietetic intervention lasting only eight weeks has very important clinical and therapeutical implications. First, this represents a mean improvement of ~1.1 mL/(kg*min) in the group of 18 T2DM participants supplemented with NO_3_^−^/NO_2_^−^, which may lead to a 9–10% decrease of all-cause mortality risk [4]. Second, cost of this simple therapeutic approach is low, which should lead to high and broad adherence in this population. Several other interesting observations arise from the present investigation.

Our findings demonstrate that the reduced NO bioavailability reported in T2DM patients, which was primarily associated with decreases in NOS-dependent NO production [17], is also associated with decreased circulating levels of NO_2_^−^, but not NO_3_^−^, when compared to age and BMI-matched controls. Individuals with several cardiovascular risk factors have been shown to present low plasma NO_2_^−^ when compared to healthier controls [40] and our results now expand these findings to type 2 diabetic patients. The mechanisms responsible for the low plasma NO_2_^−^ in T2DM remain to be established and may involve multiple processes including alterations of the oral microbiome and reduced NOS activity [41,42,43,44,45]. Nevertheless, our supplementation regimen with a daily dose of 250 mg of NO_3_^−^ and 20 mg of NO_2_^−^ for eight weeks was successful in increasing circulating levels of these NO metabolites in T2DM subjects, as previously reported [26,28]. Because the amount of NO_2_^−^ provided orally was 4–6 times lower than in previous studies targeting its direct supplementation [46,47], the dose used here can be considered relatively low and the ~40% increase of NO_2_^−^ in the circulation is likely to have resulted from both its direct supplementation and its production from supplemented NO_3_^−^. This is relevant as it suggests that larger improvements in VO_2_max may be feasible with supplementation regimens that lead to larger increases in circulating NO_2_^−^. Indeed, mean NO_2_^−^ levels in T2DM after supplementation were still at 74.6% of those in our group of non-diabetic controls. Considering the safety and feasibility of chronic NO_3_^−^ supplementation [26,28,48] and the fact that higher doses of NO_2_^−^ have been administered for longer periods such as 12 weeks [46,47], future studies examining the impact of larger doses of NO_3_^−^ and/or NO_2_^−^ (combined with a longer period of supplementation) on VO_2_max of T2DM patients are needed.

Although this novel pilot work has clinically significant indications for the management or treatment of T2DM, some aspects related to the supplementation need to be further investigated in future studies. Exercise itself is an attractive therapy for patients with T2DM, given that muscle contraction occurring with exercise can lower blood glucose independent of insulin [49] and the cardiorespiratory benefits of regular exercise in T2DM are well documented [7,50,51]. On initial screening, most subjects reported not being involved in any form of structured exercise and all participants were asked not to alter their physical activity habits during the intervention. Therefore, the potential interaction of NO_3_^−^/NO_2_^−^ supplementation and exercise needs to be addressed in more detail in future investigations. Another important point to consider in relation to study design is how other aspects of combination therapy may influence the outcomes observed. For example, supplementation of vitamin C and other antioxidants may improve the proportion of bioavailable nitrate/nitrite by inhibiting the production of potentially harmful nitrosative compounds during NO_3_^−^/NO_2_^−^ supplementation [52]. As such, follow-up studies are needed to understand if vitamin C supplementation (in combination with NO_3_^−^/NO_2_^−^) can provide additional benefits in older adults, particularly those with T2DM. Another important consideration is the potential effects of body composition changes throughout the intervention that may not be detected through assessing BMI alone. We found no differences in BMI for the primary group of T2DM subjects in the study, nor sub-group of T2DM subjects who provided muscle biopsies (Supplemental Table S1). Similar studies with nitrate or antioxidant supplementation for longer durations (10–11 weeks) in younger adults have also not found changes in body composition [53,54]. However, a potential change in body composition throughout this nutrient intervention should be investigated in future randomized controlled trials in older adults and/or T2DM patients. The potential influence of medications on the outcomes of NO_3_^−^/NO_2_^−^ supplementation also deserves attention. First, it is important to note that in the present study subjects were instructed to refrain from any medications in the morning of tests in order to avoid potential acute effects on the variables assessed. In addition, there were no differences in the proportion of subjects in the placebo and NO_3_^−^/NO_2_^−^ supplemented groups taking each medication (Table 1) and no changes were self-reported during the intervention. However, future studies examining potential interactions between NO_3_^−^/NO_2_^−^ and specific medications in T2DM are still needed. Of particular relevance to the present study, more diabetics were taking angiotensin receptor blockers (ARBs) and statin medications than non-diabetic controls. While ARBs do not seem to have deleterious effects in skeletal muscle, the effects of statins on reducing strength and integrity of skeletal muscle fibers and disrupting mitochondrial function have been documented and reviewed [55,56,57]. Still, the number of T2DM patients taking statins (or ARBs) was not different between the placebo and NO_3_^−^/NO_2_^−^ groups. Furthermore, the use of statins (or ARBs) was also not different between the highly responsive individuals in the NO_3_^−^/NO_2_^−^ group when compared to the rest of subjects in that same group. Collectively, these observations indicate that the outcomes reported from the supplementation in T2DM were not confounded by ARB or statin use. Nevertheless, whether NO_3_^−^/NO_2_^−^ supplementation can be particularly beneficial for T2DM taking statins still need to be addressed in future investigations.

Despite the relatively limited number of subjects providing muscle biopsies and the considerations outlined above, our observations on these still provide important insights into the potential mechanisms involved in the improved VO_2_max seen in T2DM patients. Decreases in VO_2_max among T2DM patients that provided muscle biopsies were accompanied by similar decreases in skeletal muscle oxidative capacity (i.e., −29.3% for VO_2_max and −26.1% and −25.5% for maximal CHO- and FA-supported respiration, respectively; Table 2 and Figure 2B,C). However, despite >90% of T2DM subjects (both in the large primary group or among those that provided muscle biopsies) presenting increases in VO_2_max after NO_3_^−^/NO_2_^−^ supplementation, only 42% of subjects that provided muscle biopsies presented improvements in maximal CHO- and FA-supported respiration. These highly responsive individuals did not present increases in CS protein when compared with the rest of the group suggesting that their superior benefits relied more on improving mitochondrial function than content (Supplemental Table S2). Additionally, the magnitudes of increase in mitochondrial respiratory capacity observed in these individuals (i.e., average of 81% and 82% under maximal CHO-supported and FA-supported respiration, respectively) were disproportionately higher than the increase in VO_2_max in this sub-group (i.e., 5.4%). Collectively, these observations indicate that those improvements in skeletal muscle oxidative capacity are not the main mechanism by which NO_3_^−^/NO_2_^−^ supplementation leads to smaller and yet consistent increases in VO_2_max of T2DM patients. Regarding this matter, supplemental oxygen has been shown to improve in vivo muscle oxidative phosphorylation in type 2 diabetics [58]. Additionally, we have recently demonstrated that forearm skeletal muscle perfusion to handgrip exercise improves ~16% after NO_3_^−^/NO_2_^−^ supplementation in the same T2DM patients studied here [26]. Given that leg muscles are actively engaged during cycle ergometry (when VO_2_max testing was performed), similar improvements in blood flow and oxygen delivery were likely to be occurring in lower limbs. Therefore, in vivo maximal skeletal muscle oxygen consumption could have increased with supplementation, thereby contributing to the improved VO_2_max observed, independently of changes in maximal mitochondrial respiratory capacity.

In summary, we report beneficial effects of combined NO_3_^−^/NO_2_^−^ supplementation for eight weeks on VO_2_max of T2DM patients, which should have important clinical implications as this may decrease all-cause mortality risks in this population. Maximal CHO- and FA-supported skeletal muscle oxidative capacity was also robustly improved in some individuals. Future studies should examine if increased doses and duration of the supplementation and, further, whether combined supplementation with an exercise regimen can lead to larger increases in VO_2_max of T2DM patients and to a larger proportion of subjects becoming responsive in terms of skeletal muscle oxidative capacity. Additionally, poor vascular function and mitochondrial dysfunction in skeletal muscle likely contribute to the high prevalence of sarcopenia reported in T2DM patients [59,60]. It will be important to examine if NO_3_^−^ and/or NO_2_^−^ supplementation can help preserve muscle mass and contractile function in this clinical population, logically extending our current findings in skeletal muscle.

## Figures and Tables

**Table 1:** Baseline clinical characteristics.

	PRIMARY GROUP				SUB-GROUP (Muscle Biopsies)			
	Control	T2DM	T2DM Placebo	T2DM NO_3_^−^/NO_2_^−^	Control	T2DM	T2DM Placebo	T2DM NO_3_^−^/NO_2_^−^
N	15	36	18	18	10	19	7	12
Age (years)	60 ± 9	59 ± 9	58 ± 9	59 ± 9	60 ± 9	60 ± 10	60 ± 12	59 ± 10
Reported duration of T2DM	- -	6 ± 4	6 ± 3	8 ± 5	- -	6 ± 4	5 ± 2	7 ± 4 ^†^
Men, n (%)	10 (67)	26 (72)	13 (72)	13 (72)	6 (60)	13 (68)	5 (71)	8 (67)
BMI (kg/m^2^)	29.5 ± 4.6	31.9 ± 5.0	32.3 ± 5.3	31.8 ± 5.0	29.4 ± 5.3	32.8 ± 5.6	32.8 ± 6.8	32.9 ± 5.5
Glucose (mg/dL)	95 ± 8	162 ± 45 ***	159 ± 40	166 ± 50	96 ± 9	184 ± 47 ***	184 ± 36	184 ± 55
Insulin (mIU/L)	14.9 ± 14.1	20.7 ± 14.3	21.7 ± 14.7	19.7 ± 14.3	12.2 ± 8.0	22.9 ± 15.7	24.7 ± 18.8	25.2 ± 18.0
HbA1c (%)	5.3 ± 0.3	7.4 ± 1.4 ***	7.3 ± 1.4	7.5 ± 1.4	5.2 ± 0.3	7.7 ± 1.4 ***	7.5 ± 1.3	7.8 ± 1.5
Prescription medications, n (%)								
Insulin	- -	10 (28)	5 (28)	5 (28)	- -	5 (26)	2 (29)	3 (25)
Metformin	- -	30 (83)	15 (83)	15 (83)	- -	17 (89)	6 (86)	11 (92)
Sulfonylurea	- -	13 (36)	5 (28)	8 (42)	- -	11 (58)	4 (57)	7 (59)
GLP-1	- -	4 (11)	2 (11)	2 (11)	- -	1 (5)	1 (14)	0 (0)
Thiazolidinediones (TZD)	- -	1 (3)	1 (6)	0 (0)	- -	1 (5)	1 (14)	0 (0)
Statin	5 (33)	26 (72) ***	12 (67)	14 (78)	2 (20)	12 (74) *	4 (57)	8 (67)
ACE Inhibitor (ACEi)	2 (13)	11 (31)	5 (28)	6 (21)	1 (10)	7 (37)	3 (43)	3 (25)
Angiotensin Receptor Blocker (ARB)	0 (0)	9 (25) *	4 (22)	5 (28)	0 (0)	5 (26) ^#^	2 (29)	3 (25)
Beta-blocker	2 (13)	8 (22)	4 (22)	4 (22)	1 (5)	3 (16)	1 (14)	2 (17)
Ca^2+^ Channel blocker	2 (13)	3 (10)	2 (11)	1 (6)	1 (5)	2 (11)	1 (14)	1 (8)
Hydrochlorothiazide (HCTZ)	0 (0)	4 (13)	3 (17)	1 (6)	0 (0)	2 (11)	1 (14)	1 (8)

Data are presented as mean ± standard deviation. T2DM, Type 2 Diabetes Mellitus. NHbA1c, glycosylated hemoglobin. GLP-1, glucagon-like peptide-1. * *p* < 0.05, *** *p* < 0.001 and ^#^ *p* = 0.07 vs. respective non-diabetic control group. ^†^ *p* < 0.05 vs. Placebo in the Sub-group.

**Table 2:** Baseline plasma NO metabolites and cardiorespiratory fitness.

	PRIMARY GROUP				SUB-GROUP (Muscle Biopsies)			
	Control	T2DM	T2DM Placebo	T2DM NO_3_^−^/NO_2_^−^	Control	T2DM	T2DM Placebo	T2DM NO_3_^−^/NO_2_^−^
N	15	36	18	18	10	19	7	12
Plasma Nitrate, NO_3_^−^(μM)	12.8 ± 5.0	25.6 ± 13.7 ***	27.2 ± 11.1	23.9 ± 16.0	11.4 ± 2.9	24.8 ± 15.8 ***	24.4 ± 11.8	25.0 ± 19.0
Plasma Nitrite, NO_2_^−^(μM)	0.48 ± 087	0.26 ± 0.11 ****	0.26 ± 0.13	0.26 ± 0.09	0.45 ± 0.08	0.26 ± 0.11 ***	0.28 ± 0.13	0.25 ± 1.00
VO_2_max (ml/(kg*min))	27.6 ± 8.7	20.3 ± 5.7 ***	20.0 ± 4.2	20.7 ± 7.0	28.3 ± 7.1	20.0 ± 6.0 **	18.5 ± 1.1	20.0 ± 6.8
Work Rate (Wmax)	178 ± 46	136 ± 40 **	138 ± 32	135 ± 47	186 ± 48	130 ± 30 ***	129 ± 25	127 ± 33

Data are presented as mean ± standard deviation. ** *p* < 0.05, *** *p* < 0.001 and **** *p* < 0.0001 vs. respective non-diabetic control group.

## Data Availability

The data presented in this study are available on request from the corresponding author.

## Supplementary Materials

The following supporting information can be downloaded at: https://www.mdpi.com/article/10.3390/nu14214479/s1, Figure S1: Citrate Synthase (CS) expression in T2DM patients and non-diabetic controls; Figure S2: Plasma nitrate (NO_3_^−^) and nitrite (NO_2_^−^ ) levels after combined NO_3_^−^/NO_2_^−^ supplementation vs. Placebo in T2DM participants that provided muscle biopsies (Sub-group); Figure S3: Impact of combined nitrate/nitrite (NO_3_^−^/NO_2_^−^) supplementation on maximal oxygen uptake capacity (VO_2_max) in T2DM participants that provided muscle biopsies (Sub-group); Figure S4: Changes in skeletal muscle mitochondria respiratory capacity and citrate synthase expression resulting from combined NO_3_^−^/NO_2_^−^ supplementation in T2DM participants; Table S1: Clinical post-supplementation characteristics of T2DM subjects that provided muscle biopsies (Sub-group); Table S2: Baseline characteristics and delta changes with supplementation of highly responsive (in terms of skeletal muscle oxidative capacity) vs. little or not responsive T2DM subjects in the NO_3_^−^/NO_2_^−^ group that provided muscle biopsies (Sub-group).
