# Supplementary material for: Effects of Combined Inorganic Nitrate and Nitrite Supplementation on Cardiorespiratory Fitness and Skeletal Muscle Oxidative Capacity in Type 2 Diabetes: A Pilot Randomized Controlled Trial
Source: Nutrients. 2022 Oct 25;14(21):4479. doi: 10.3390/nu14214479 (PMC9654804; doi:10.3390/nu14214479)
Supplement: Supplementary file 1 [file nutrients-14-04479-s001.zip › nutrients-1975657-SI.pdf]

Effects of Combined Inorganic Nitrate and Nitrite Supplementation on Cardiorespiratory Fitness and Skeletal Muscle Oxidative Capacity in Type 2 Diabetes: A Pilot Randomized Controlled Trial

Kristen D. Turner [1], Ana Kronemberger [1], Dam Bae [1], Joshua M. Bock [2], William E. Hughes [2], Kenichi Ueda [3], Andrew J. Feider [3], Satoshi Hanada [3], Luis G. O. de Sousa [4], Matthew P. Harris [1], Ethan J. Anderson [5,6,7], Sue C. Bodine [4,6], M. Bridget Zimmerman [8], Darren P. Casey [2,6,7,9] and Vitor A. Lira [1,6,7,9,*]

SUPPLEMENTARY MATERIAL

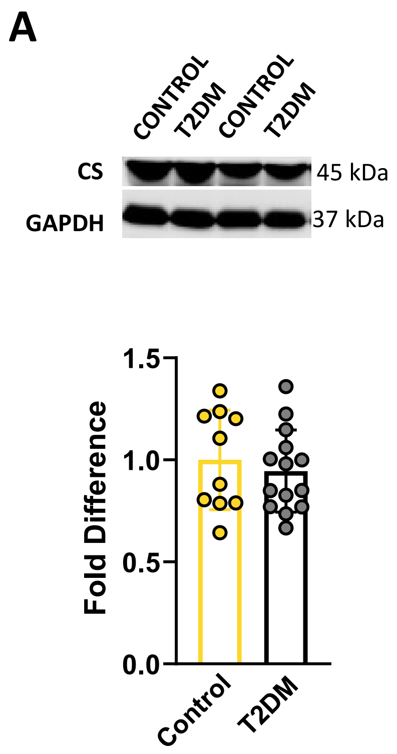

Supplemental Figure S1. Citrate Synthase (CS) expression in T2DM patients and non-diabetic controls. A) (Top) Representative western blots for CS protein. (Bottom) Quantification of CS protein expression in muscles from controls (N=10) and T2DM subjects (N=14). Data are presented as mean±SD.

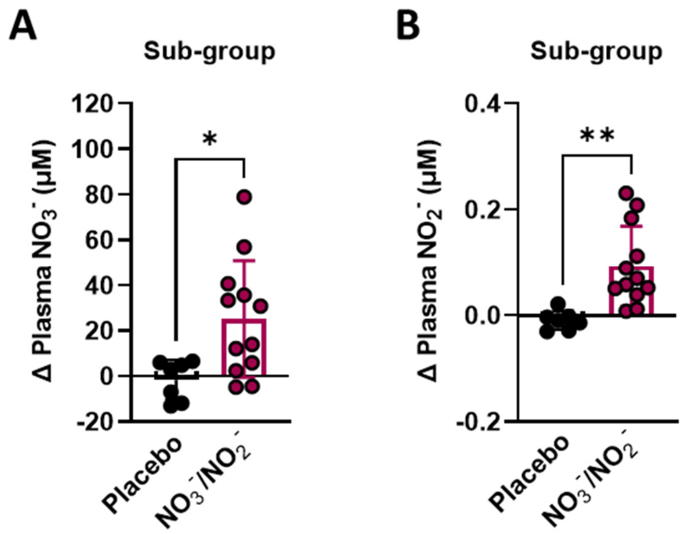

Supplemental Figure S2. Plasma nitrate (NO_3^-) and nitrite (NO_2^-) levels after combined NO_3^-/NO_2^- supplementation vs. Placebo in T2DM participants that provided muscle biopsies (Sub-group). A) ANCOVA results, using pre-supplementation values of NO_3^- as a covariate, showing individual changes (post – pre) for plasma NO_3^- from T2DM subjects (N=7 for placebo and N=12 for NO_3^-/NO_2^- group). B) ANCOVA results, using pre-supplementation values of NO_2^- as a covariate, showing individual changes (post – pre) for plasma NO_2^- as shown in A (N=7 for placebo and N=12 for NO_3^-/NO_2^- group). Data are presented as mean±SD. *$P < 0.05$; **$P<0.01$.

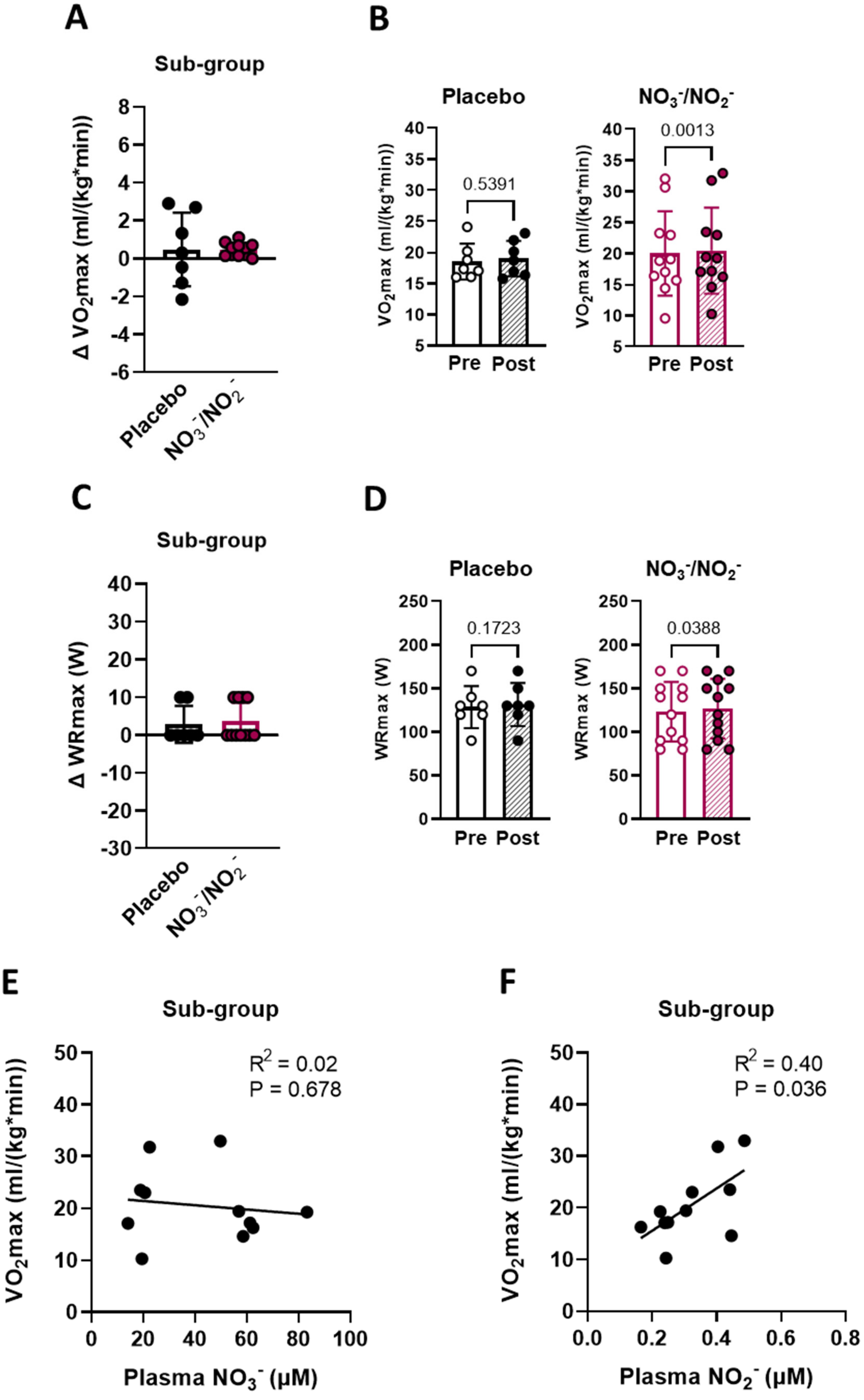
A
B
C
D
E
F
Sub-group
Placebo
NO_3^-/NO_2^-
Δ VO_2max (ml/(kg*min))
VO_2max (ml/(kg*min))
Pre
Post
0.5391
0.0013
Δ WRmax (W)
WRmax (W)
0.1723
0.0388
R^2 = 0.02
P = 0.678
R^2 = 0.40
P = 0.036
Plasma NO_3^- (µM)
Plasma NO_2^- (µM)

Supplemental Figure S3. Impact of combined nitrate/nitrite (NO_3^-/NO_2^-) supplementation on maximal oxygen uptake capacity (VO_2max) in T2DM participants that provided muscle biopsies (Sub-group). A) ANCOVA results, using pre-supplementation values of VO_2max as a covariate, showing individual changes (post – pre) for VO_2max from sub-groups of T2DM subjects (N=7 for placebo and N=12 for NO_3^-/NO_2^- group). B) Paired T-tests for VO_2max before and after intervention from sub-groups of T2DM subjects (N=7 for placebo and N=12 for NO_3^-/NO_2^- group). C) ANCOVA results, using pre-supplementation values of maximal work rate (WRmax) as a covariate, showing individual changes (post – pre) for WRmax from sub-groups of T2DM subjects (N=7 for placebo and N=12 for NO_3^-/NO_2^- group). D) Paired T-tests for WRmax before and after intervention from sub-groups of T2DM subjects (N=7 for placebo and N=12 for NO_3^-/NO_2^- group). E) Relationship between plasma NO_3^- and VO_2max in the sub-group of T2DM subjects (N = 12; r = 0.09; Slope 95% CI: -0.257, 0.175). B) Relationship between NO_2^- and VO_2max in the sub-group of T2DM subjects (N = 12; r = 0.64; Slope 95% CI: 3.343, 78.36).

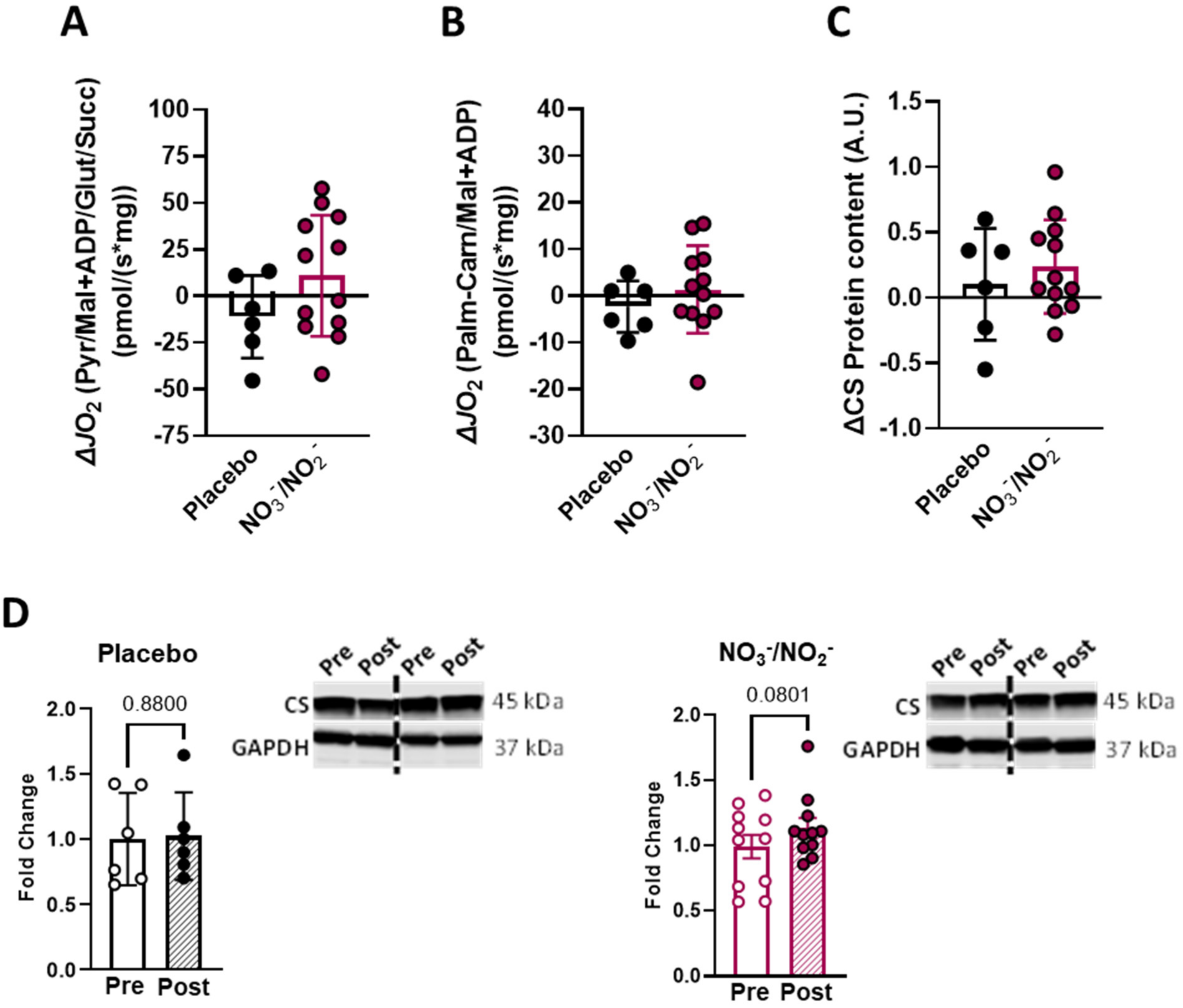

Supplemental Figure S4. Changes in skeletal muscle mitochondria respiratory capacity and citrate synthase expression resulting from combined NO_3^-/NO_2^- supplementation in T2DM participants. A) ANCOVA results, using pre-supplementation values of JO_2 as a covariate, showing mean individual delta changes (post – pre) for JO_2 under maximal CHO-supported respiration (N=7 for placebo and N=12 for NO_3^-/NO_2^- group) (P=0.16). B) ANCOVA results, using pre-supplementation values of JO_2 as a covariate, showing mean individual delta changes (post – pre) for JO_2 under maximal FA-supported respiration (N=7 for placebo and N=12 for NO_3^-/NO_2^- group) (P=0.39). C) ANCOVA results, using pre-supplementation values of CS as a covariate, showing mean individual delta changes (post – pre) for CS (N=6 for placebo and N=12 for NO_3^-/NO_2^- group) (P=0.49). D) Paired T-tests and representative western blots for CS before and after intervention for the Placebo group (Left, N=6) and NO_3^-/NO_2^- group (Right, N=12). Data are presented as mean±SD.

Supplemental Table S1. Clinical post-supplementation characteristics of T2DM subjects that provided muscle biopsies (Sub-group).

	SUB-GROUP			
	**T2DM Placebo (post)**	**T2DM Placebo (% change vs. pre)**	**T2DM NO_3^-/NO_2^- (post)**	**T2DM NO_3^-/NO_2^- (% change vs. pre)**
N	7	- -	12	- -
Age (years)	60±12	- -	58±10	- -
BMI (kg/m^2)	32.5±6.3	-0.7±3.1	33.0±5.5	0.4±2.5
Glucose (mg/dL)	171±49	-11±23	185±65	-4±27
Insulin (µU/mL)	22.2±15.7	-9±36	25.2±18.0	-1±45
HbA1c (%)	7.5±1.7	-1.3±12.1	7.7±1.2	-2.0±10.5

Data are presented as mean±standard deviation. No significant differences were observed across groups or in comparison to pre-supplementation values.

Supplemental Table S2. Baseline characteristics and delta changes with supplementation of highly responsive (in terms of skeletal muscle oxidative capacity) vs. little or not responsive T2DM subjects in the NO_3^-/NO_2^- group that provided muscle biopsies (Sub-group).

	**NO_3^-/NO_2^- supplementation (mitochondrial respiration)**		
	**Highly Responsive**	**Others**	**P**
N	5	7	--
Age (years)	56.2±10.89	60.3±9.23	0.50
Men, n (%)	3 (60)	5 (71)	0.68
BMI (kg/m^2)	32.7±7.88	32.9±2.77	0.97
Glucose (mg/dL)	154.2±42.12	190.4±53.66	0.24
Insulin (μU/mL)	26.5±17.54	19.0±11.26	0.39
HbA1c (%)	7.1±1.29	8.4±1.40	0.12
NO_3^- (μM)	18.6±4.9	29.6±24.2	0.34
Δ NO_3^- (μM)	15.3±17.5	32.2±29.7	0.29
NO_2^- (μM)	0.3±0.18	0.2±0.07	0.45
Δ NO_2^- (μM)	0.10±0.08	0.09±0.08	0.77
VO_2max (ml/(kg*min))	22.55±8.7	17.65±3.2	0.22
Δ VO_2max (ml/(kg*min))	1.21±1.13	0.31±0.24	0.09
Citrate Synthase (CS) protein (A.U.)	1.27±0.28	1.10±0.36	0.41
Δ CS protein (A.U.)	0.37±0.38	0.17±0.32	0.43
Prescription Medications, n (%)			
Insulin	1 (20)	2 (29)	0.74
Metformin	4 (80)	7 (100)	0.22
Sulfonylureas	3 (60)	4 (57)	0.92
Statins	4 (80)	4 (57)	0.41
ACE inhibitors	1 (20)	2 (29)	0.74
Angiotensin Receptor Blocker (ARB)	2 (40)	1 (14)	0.31
Beta-blocker	2 (40)	0 (0)	0.07

Data are presented as mean±standard deviation. A.U. – arbitrary units.

Supplemental Material and Methods

Western Blots

Muscle lysates were obtained as previously described [1], and citrate synthase protein was normalized to GAPDH. Antibodies used were citrate synthase (SAB2701077, Sigma; 1:2000 dilution) and GAPDH (2118, Cell Signaling; 1:2000 dilution). All analyses were carried out using Image Lab Software 6.0.1 (Bio-Rad, USA).

Reference:

[1] Fuqua JD, Mere CP, Kronemberger A, Blomme J, Bae D, Turner KD, et al. ULK2 is essential for degradation of ubiquitinated protein aggregates and homeostasis in skeletal muscle. FASEB J 2019;33:11735–2745. https://doi.org/10.1096/FJ.201900766R.
